# Autonomic dysfunction detected by skin sympathetic response in Lambert-Eaton myasthenic syndrome: a case report

**DOI:** 10.1186/s12883-022-02625-1

**Published:** 2022-03-19

**Authors:** Jinghong Zhang, Xusheng Huang, Qiang Shi

**Affiliations:** Department of Neurology, the First Medical Centre, Chinese PLA General Hospital, Beijing, China

**Keywords:** Lambert-Eaton myasthenic syndrome (LEMS), Autonomic dysfunction, Skin sympathetic response (SSR)

## Abstract

**Background:**

Lambert-Eaton myasthenic syndrome (LEMS) is a type of paraneoplastic syndrome that may initially manifest itself with proximal weakness and gait abnormalities. Approximately up to 50% of LEMS patients have a primary autonomic dysfunction.

**Case presentation:**

We present here a case of a 75-year-old male with symmetric proximal muscle weakness, dry mouth and constipation. The cutaneous response to scratch and upright tilt-table testing were positive. A repetitive nerve stimulation test showed that there was a decremental response of compound muscle action potential (CMAP) amplitude at 3 Hz while an incremental response at 20 Hz. The presence of antibodies against voltage-gated calcium channels (VGCC) confirmed the diagnosis. Because of the prominent symptom of autonomic disorder, the patient further underwent the test of skin sympathetic response (SSR). Lower amplitude and longer response duration were found in palms, while it evoked no response in soles.

**Conclusions:**

In this case, we present the detailed results of SSR test on a patient suffering LEMS with autonomic disorder. Since autonomic dysfunction has a significant impact on clinical management and SSR test is an effective detection method, we recommend that SSR test be performed on patients with LEMS regularly.

## Background

Lambert-Eaton myasthenic syndrome (LEMS), first reported by Eaton L M and Lambert E H in 1957 [1], is a type of paraneoplastic syndrome that may initially manifest itself with proximal weakness and gait abnormalities. Several studies have reported cases of autonomic dysfunction in LEMS [2–5]. Here in this paper, we report a case of Lambert-Eaton syndrome with autonomic dysfunction confirmed by SSR test.

## Methods

The electrodiagnostic studies, including skin sympathetic response (SSR) and repetitive nerve stimulation (RNS), were performed on Cadwell’s Sierra Summit. Both SSR and RNS tests were recorded in a warm room kept at 24 °C or higher, with the skin temperature at 32 °C or above [6].

The SSR procedure involved 10 mm disk electrodes firmly attached to the volar and dorsal surfaces of the hands and feet. Median nerve and posterior tibial nerve are stimulated by electric shocks. The stimulus duration was 0.2 ms with the intensity among 10 mV to 30 mV. To avoid habituation, stimuli were administered at irregular long intervals (more than 1 min). The latency was measured from the onset of the stimulus artifact to first deflection from baseline. The amplitude was measured from the basal line to the peak of first negative or positive deflection. A response was defined as absent if no reproducible deflection could be recorded after three times of consecutive and irregular stimulation. With reference to the normative values set up by EMG laboratory of Peking Union Medical College Hospital, the normal values of latency and amplitude for hands and feet are as follows: hands (lantency:1044-1508 ms, amplitude: 1400-4560 μV); feet (latency:1403-2449 ms, amplitude: 500-1820 μV). Absent response and response with longer latency or lower amplitude are considered abnormal.

For RNS test, surface electrodes were used to record the belly-tendon compound muscle action potential (CMAP). RNS was performed in the following muscles: abductor pollicis brevis for the median nerve, abductor digiti minimi for the ulnar nerve, tibialis anterior for the common peroneal nerve. The peak-to-peak CMAP amplitude decrement was measured by the decremental percentage of the forth CMAP as compared to the first CMAP amplitude. Based on the conventional criterion, both a decremental response of 10% or greater in 1 Hz or 3 Hz and an incremental response of 100% or greater in 10 Hz or 20 Hz were considered positive, in accordance with the suggestions of the American Academy of Emergency Medicine Quality Assurance Committee.

## Case presentation

**Fig. 1:**
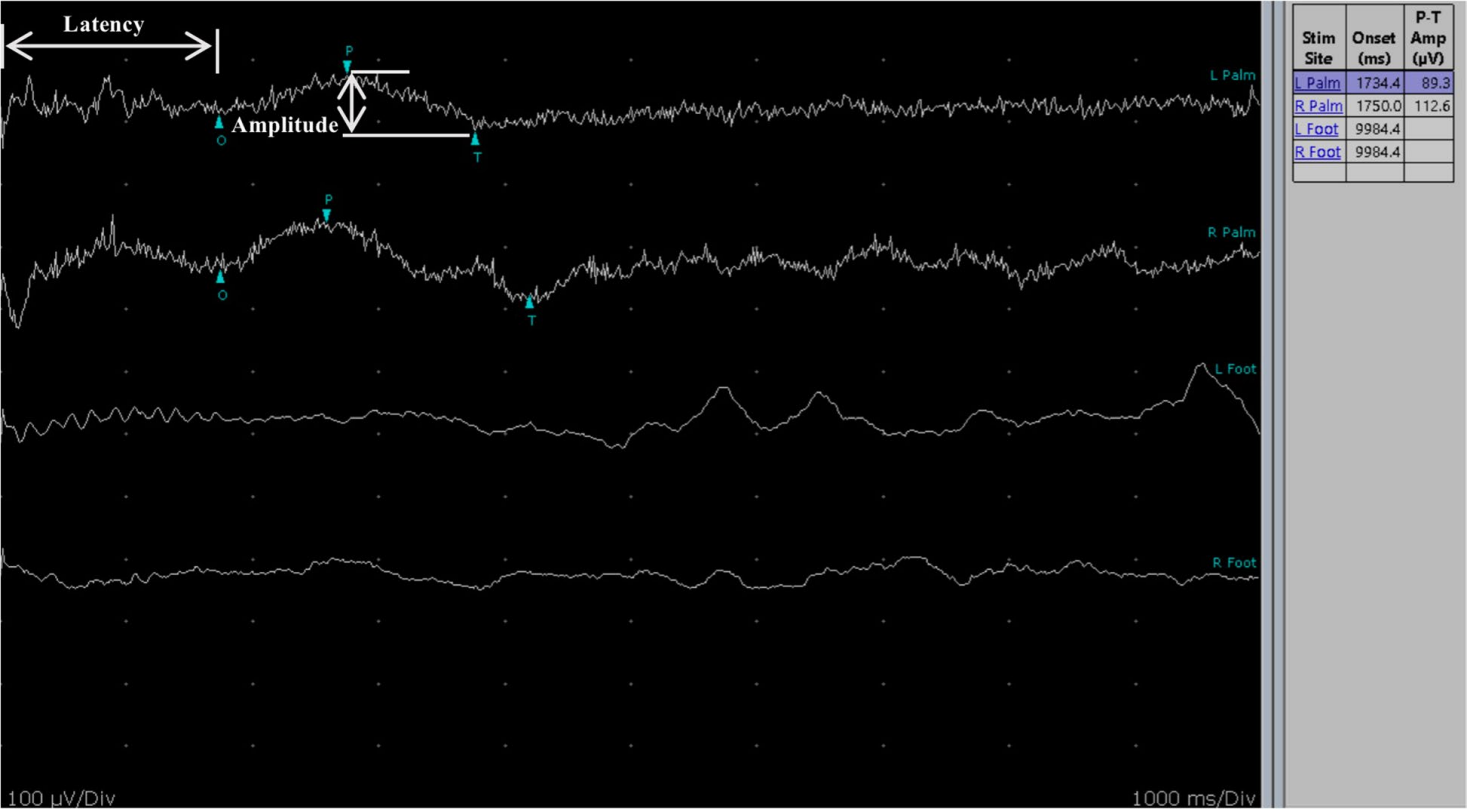
Sympethetic skin response (SSR) recorded from bilateral palms and feet detected lower amplitude and longer latency in palms, and no response was evoked in soles

A 75-year-old male with no special medical history was inflicted with dry mouth and constipation for 2 months, and symmetric proximal muscle weakness for 1 month. The muscle strength of the both proximal limbs was 4/5, and that of the distal limbs 5/5. There was no sign of tendon reflexes in all the limbs while the reflex of the right knee increased to 1+ after a sustained 30-s contraction. Notably, the cutaneous response to scratch was positive. Orthostatic hypotension was found by upright tilt-table testing. Chest computed tomography, brain magnetic resonance imaging, ultrasounds of abdomen and lymph nodes, and serum tumor markers showed absolutely no evidence of tumor. Electromyography showed no neurogenic or myogenic damage, and the conduction velocity of the sensory nerve and motor nerve was normal. Repetitive nerve stimulation (RNS) at 3 Hz on the right common peroneal nerve resulted in 30% decrement of the compound muscle action potential (CMAP) amplitude while that on the left common peroneal nerve reached 32% (Table 1). 20 Hz RNS test showed large increment of CMAP amplitude on bilateral common peroneal nerves (left one: 164%; right one: 112%). Voltage-gated calcium channels (VGCC) antibody was positive while anti-SRY-Related HMG-Box Gene 1(SOX1) antibody was negative. All considered, the patient was diagnosed with Lambert-Eaton syndrome. Because of the prominent symptom of autonomic disorder, the patient further underwent skin sympathetic response (SSR) test so as to confirm the autonomic dysfunction (Fig. 1, Table 2). Longer latency and lower amplitude were detected on bilateral palms in the first SSR, while it evoked no response in the sole after three times of stimulation. The patient received a five-day treatment by intravenous immunoglobulins (0.4 g/kg/d). Until now, the patient has been followed-up for 6 months. Recently, he suffers from muscle weakness and difficulties in walking. But the symptoms of dry mouth and constipation do not occur again. The chest CT and blood tests for malignancy are performed every 3 months and the results show that he remains cancer-free.

**Table 1:** The results of RNS test

Tested nerves	1 Hz	3 Hz	10 Hz	20 Hz
Left median nerve	↓18%	↓27%	↓16%	↑45%
Left ulnar nerve	↓7%	↓13%	↓1%	↑98%
Left common peroneal nerve	↓27%	↓39%	↓32%	↑164%
Right common peroneal nerve	↓19%	↓30%	↓34%	↑112%

“↓” indicates decremental response. “↑” indicates incremental response

**Table 2:** The results of SSR test

Tested body part	Latency (ms)	Normal range of lantency (ms)	Amplitude (μV)	Normal range of amplitude (μV)
Left palm	1734	1044-1508	89	1400-4560
Right palm	1750		112	
Left sole	–	1403-2449	–	500-1820
Right sole	–		–	

“-” indicates absent response

## Discussion and conclusions

The Lambert-Eaton myasthenic syndrome is a neuromuscular junction disorder characterized by proximal weakness and autonomic dysfunction. Since autonomic dysfunction can not only cause diverse uncomfortable symptoms, but also lead to cardiac arrhythmias and even sudden death, many researches have focused on the range of autonomic symptoms and signs occuring in them [2, 4, 5, 7, 8].

Approximately up to 50% of LEMS patients have a primary autonomic dysfunction including dry mouth, constipation, loss of sweating, orthostatic hypotension, pupillary abnormalities etc [4, 7, 8] In the assessment of the autonomic function in patients with LEMS, previous studies mainly focus on the results of following tests: heart rate (HR) responses to tilting/ deep breathing and Valsalva maneuver/cold face test, blood pressure (BP) responses to tilting/ Valsalva maneuver (systolic BP overshoot)/mental arithemic/noradrenaline infusion, Valsalva ratio, test of sweating (including sweat response to thermal stimulation and quantitative sudomotor axon reflex test), etc [2] In our case, a history of obvious dry mouth and constipation at an early stage with abnormalities in tilt-table testing and cutaneous response to scratch is a compelling reminder of autonomic dysfunction. SSR test electrophysiologically confirms the involvement of autonomic nervous system.

SSR, a non-invasive measure to assess sympathetic cholinergic sudomotor function, has not been widely performed on LEMS patients. To date, only two case reports [9, 10] have mentioned SSR test on LEMS patients, and one study suggests the absence of SSR in 18 out of 33 cases [11]. None of them describe the detailed results of the test as we do in the current presentation. Besides, it’s reported that SSR test not only provides a quantitative functional measure of sudomotor activity [12], but also has the potential to detect subclinical autonomic nervous system dysfunction even in patients who do not have autonomic complaints [13]. It is worth noting that in our case, the absence of SSR is not accompanied by any complaint of sweat disturbance, which suggests that the measurement of SSR might represent subclinical symptom. As it is, the relationship between sweat disturbance and the positivity rate of SSR test needs further investigation.

Until recently, little has been known about the mechanisms underlying autonomic dysfunction in immune-related neuromuscular junction diseases. Several studies have demonstrated the possible mechanism in myasthenia gravis (MG) patients: in anti-acetylcholine receptor (AChR) antibody (Ab)-positive MG patients, the presence of autonomic dysfunction could be the result of the cross reactivity of muscular anti-AChR Ab with ganglionic AChR [14], whereas in anti-muscle-specific kinase (MuSK) antibody-positive MG patients, autonomic failure may related to the antibodies against agrin, lipoprotein receptor-related protein 4(Lrp4) and other immunological targets on the neuromuscular junction [15]. Nalbantoglu et al [16] also suggest that acetylcholinesterase inhibitors might trigger autonomic symptoms as they extend the life span of acetylcholine secreted from the cholinergic nerve endings and thus accumulate in the synaptic gap, leading to the excitation of all the cholinergic receptors including muscarinic and nicotinic receptors of autonomic nervous system. Furthermore, autoantibody seems not only a factor contributing to autonomic dysfunction in MG, but also in LEMS. P/Q type voltage-gated calcium channel (VGCC) antibodies are present in 80 to 90% of patients with LEMS [17, 18]. Taking the high positivity rate of P/Q VGCC antibodies as well as the high incidence of autonomic dysfunction in patients with LEMS into consideration, the relationship between them needs to be investigated. Sally [2] suggests that autoantibodies to the P/Q-subtype of VGCC might inhibit transmitter release from parasympathetic, sympathetic, and enteric neurons in LEMS patients. Vincent [19] points out that the autonomic symptoms in LEMS is due to disturbance of acetycholine release at smooth muscle, as a result of the increased internalisation and degradation of VGCC by divalent antibodies’ cross-linking of ion channel. Although the relationship between VGCC antiobodies and autonomic dysfunction in LEMS is still unclear, the study of Wan et al [20] confirms that VGCC autoantibodies in type 1 diabetes mediate autonomic dysfunction of the gastrointestinal tract and bladder, and IVIg has a direct neutralizing effect on anti-VGCC Abs in vivo. Their findings enlighten the underlying mechanism for the disappearance of dry mouth and constipation in our patient after a five-day treatment by IVIg, indicating that IVIg might improve autonomic dysfunction in LEMS. Therefore, this case provide a rationale for investigating the effect of IVIg for autonomic dysfunction in LEMS patients with VGCC antibodies.

In conclusion, in this case, we present the detailed results of SSR test on a patient suffering LEMS and autonomic disorder simultaneously. Since SSR test is an effective way to detect autonomic dysfunction, we recommend that SSR test be performed on patients with LEMS regularly.

## Data Availability

All data related to this case report are documented within this manuscript.

## Abbreviations

LEMS: Lambert-Eaton myasthenic syndrome
CMAP: Compound muscle action potential
VGCC: Voltage-gated calcium channels
SSR: Skin sympathetic response
RNS: Repetitive nerve stimulation
SOX1: Anti-SRY-Related HMG-Box Gene 1
MG: Myasthenia gravis
HR: Heart rate
BP: Blood pressure
HRV: Heart rate variability
IVIg: Intravenous immunoglobulins
AChR: Acetylcholine receptor
Ab: Antibody
Lrp4: Lipoprotein receptor-related protein 4
MuSK: Muscle-specific kinase
